# Unravelling three-way interactions between Clostridioides difficile, microbiota and the host

**DOI:** 10.1099/jmm.0.002067

**Published:** 2025-10-03

**Authors:** Kavana K. Bywater-Brenna, Meera Unnikrishnan

**Affiliations:** 1 Division of Biomedical Sciences, Warwick Medical School, University of Warwick, Coventry, UK

**Keywords:** *Clostridioides difficile *infection, host–microbiome interactions, host–pathogen interactions, pathogenesis

## Abstract

*Clostridioides difficile* infection is a global issue, representing a huge financial burden on healthcare systems worldwide which is further exacerbated by high recurrence rates. Infection is closely linked with the gut microbiome status, with successful *C. difficile* colonization usually occurring when there is dysbiosis. Our understanding of the molecular mechanisms underlying microbiota-mediated colonization resistance has advanced significantly in recent years, although the nuanced crosstalk occurring between *C. difficile*, the gut microbiota and host mucosa has yet to be fully elucidated. Deciphering these three-way interactions is critical for the development of effective therapeutic and prophylactic strategies. This review will discuss known interactions between this pathogen, the microbiota and the host in addition to the tools available to dissect complex microbial interchanges.

## Introduction

The gut microbiota is central to human health and has been linked to several metabolic and infectious conditions. *Clostridioides difficile* infection (CDI), an infection characterized by serious diarrhoea and pseudomembranous colitis (PMC), is one such condition that has been closely associated with the gut microbiota [1,2]. Antibiotic therapy which results in the disruption of the gut microbiota is a major risk factor for CDI [3,4]. CDI represents one of the most common hospital-associated infections to date, with an incidence of 8.3 cases of hospital-onset CDI per 10,000 patient days [5,6]. Recurrence presents a significant challenge to the successful treatment of infection, with recurrence following a successful primary treatment occurring in 20–35% of patients [7]. High recurrence rates have been associated with high healthcare costs across the world. Estimated annual economic burdens vary, estimated between $5.6–6.3 billion in the USA and €3 billion in the European Union, with infections expected to double over the coming four decades [7,11]

*C. difficile* is a spore-forming obligate anaerobic pathogen and aetiological cause of CDI. The role a healthy gut microbiota plays in preventing *C. difficile* spore germination and proliferation has been well documented, with several studies highlighting the strong association of CDI with distinct changes in gut microbiota composition [12,14]. In the last decade, studies have also revealed exciting insights into the mechanisms underlying colonization resistance against CDI [15,18]. In this review, we will discuss *C. difficile*–microbiota interactions at a population and single species level and further examine the complex three-way interactions between *C. difficile*, the gut microbiota and the host immune system. We will also discuss tools that are being employed to probe these highly intricate interactions.

## The gut microbiota and colonization resistance

More than 10^13^ micro-organisms are thought to populate the gastrointestinal tract, 100-fold more bacteria than in any other microbial population in the human body, and although they were originally thought to outnumber the total gut host cells 10 : 1, this ratio is now considered to be closer to 1 : 1 [19,21]. Metagenomic studies have yielded great insight into the microbial architecture within the gut, showing that it is dominated by four main phyla: *Bacteroidetes*, *Firmicutes*, *Actinobacteria* and *Proteobacteria* [22,23]. The composition of the gut community is known to vary across the gut, and how such differences give rise to distinct microbiota compositions has been previously reviewed [24,25]. *C. difficile* primarily colonizes the large intestine, where *Bacteroidetes* and *Firmicutes* account for over 90% of the gut microbiota in the distal region. However, vegetative *C. difficile* cells can persist throughout the gastrointestinal tract during prolonged infections [26,27].

**Fig. 1.**
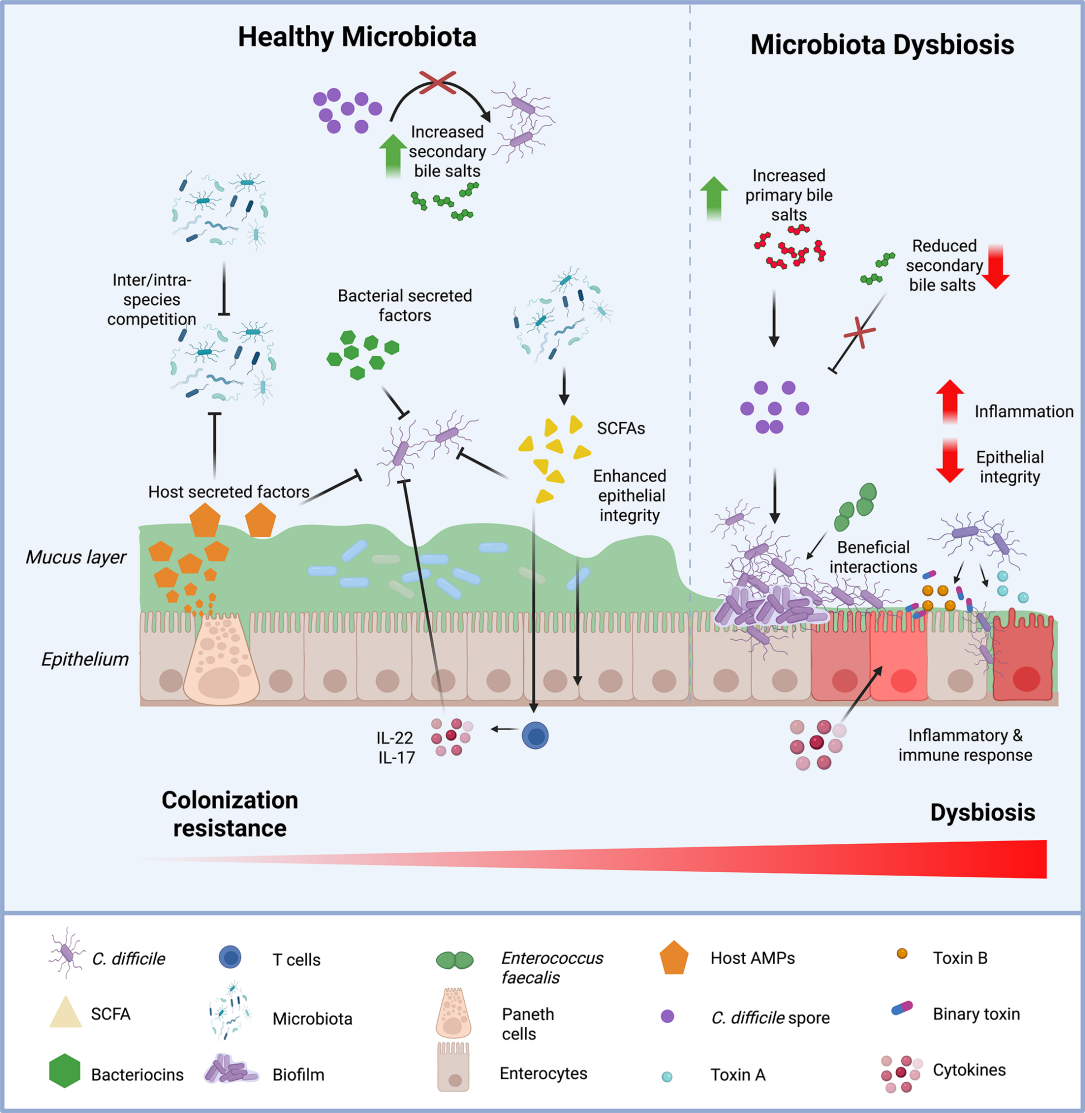
Colonization resistance and CDI. The microbiota plays multiple important roles in conferring colonization resistance against *C. difficile*. This can be through the production of inhibitory metabolites, through the conversion of primary bile salts to secondary bile salts, which are inhibitory to *C. difficile* spore germination. Production of SCFAs can also inhibit *C. difficile* but can also modulate the immune system, leading to the production of IL-17 and IL-22, for example, whilst also helping to maintain the epithelial integrity. In addition to immune responses, the host produces secreted factors such as inhibitory antimicrobial peptides (AMPs) which help to maintain the microbiota homeostasis but also inhibit *C. difficile*. Perturbations in the composition of the gut cause a change in the dominant members, leading to a loss of colonization resistance, often characterized with a loss of *Firmicutes* abundance, with increased *Lactobacillaceae*, *Proteobacteria* and *Enterobacteriaceae*. The loss of many commensals means there are more primary bile salts present, leading to the germination of *C. difficile* spores and subsequent outgrowth, followed by toxin production. The action of toxins causes the loss of epithelial barrier integrity, in addition to a large inflammatory response by the host. Additionally, enterococcal enrichment, a likely outcome of gut perturbation, also facilitates certain beneficial interactions between specific enterococcal species and *C. difficile*. Figure was generated using BioRender.com.

One of the main functions of the gut microbiota is to act as a critical line of defence, resisting the invasion and expansion of pathogens, which is broadly termed colonization resistance [28] (Fig. 1). Higher diversity microbiomes are associated with increased colonization resistance and the microbiota circumvents infection via a range of mechanisms such as nutrient and space competition, short-chain fatty acid (SCFA) production, bile salt metabolism, antimicrobial compound production and immune modulation of the host [29,30]. Such mechanisms can be categorized into either direct or indirect mechanisms: the former involves host-independent bacterium–bacterium interactions, whilst the latter relates to host–bacterium interactions [31]. Direct mechanisms are twofold, either involving ‘interference’ competition via the production of inhibitory compounds or through ‘exploitative’ competition via resource and niche competition [32].

## *C. difficile* interactions with the microbiota

The notion that the resident gut microbiota could confer some kind of resistance against bacterial pathogen colonization existed well before the establishment of an association between *C. difficile* and antibiotic-mediated PMC [33]. In 1974, a large-scale study of clindamycin-associated colitis reported that clindamycin administration was associated with diarrhoea and PMC in 21% and 10% of patients, respectively [34]. It was not, however, until 1977 that Bartlett *et al.* first proposed that a toxin-producing *Clostridium* species, at the time named *Clostridium BVA 17 HF1-9*, was the cause of clindamycin-associated PMC [35]. The role of *C. difficile* as the causative micro-organism was shortly thereafter confirmed through the fulfilment of Koch’s postulates in a hamster model [36]. Following this, *C. difficile* toxins were implicated as a major aetiological cause of clindamycin-associated PMC [37,38], and CDI has also gone on to be strongly associated with other antibiotic usage, such as the broad-spectrum antibiotics third-generation cephalosporins and fluoroquinolones [3]. Whilst it is well established that the toxins are responsible for many of the clinical manifestations of CDI, it is increasingly recognized that the spores of *C. difficile*, rather than the toxins alone, are the primary drivers of CDI, acting as the critical vehicle of disease [38,39].

A plethora of studies looking more closely at *C. difficile* interactions with the resident microbiota have been conducted in recent years, and specific antibiotic-driven perturbations of the gut microbiota have been associated with CDI development, outcome and risk [2,40]. Murine models have been instrumental in establishing that antibiotic administration results in shifts in the composition of the gut microbiota, as well as in identifying an interplay between ecological dynamics and how the severity of *C. difficile*-associated colitis can be attenuated or aggravated through alterations in gut composition [41,43]. Such models have provided informative insights showing CDI is associated with a decrease in overall diversity, characterized by a shift in the dominating phylum, from *Firmicutes* to *Lactobacillaceae*, when given a five-antibiotic cocktail [42]. An apparent bias in dysbiosis has also been observed, whereby shifts in the microbiota are distinct and antibiotic class-specific, with clindamycin alone resulting in a profile shifted towards *Proteobacteria* and *Enterobacteriaceae* dominance [42], and beta-lactams favouring enterococcal enrichment [44].

Longitudinal associations on hospitalized patients have also demonstrated that patients developing CDI presented with significantly less microbial abundance combined with distinct microbiota compositions before antibiotic application, enriched in *Enterococcus* and depleted of *Ruminicoccus*, *Bifidobacterium*, *Prevotella* and *Blautia*, suggesting how changes in gut microbiota structure may prime or promote development of CDI [44]. This associative link between microbiota and CDI was further supported through the restorative properties of faecal microbiota transplants (FMTs), which have been used as a treatment for PMC dating back to 1958 [45]. The use of FMTs has been shown to restore the gut composition to levels reminiscent of microbiota derived from healthy donor samples [46], whilst also successfully restoring bile salt levels to pre-challenged conditions [47]. Studies employing *in vitro* communities have shown that the *C. difficile* growth is heavily influenced by gut species richness [48].

### The role of bile salts on *C. difficile* germination

One of the main ways the microbiota interacts with *C. difficile* is by preventing spore germination in the first instance – a crucial first step in colonization. The stimulatory effect of certain primary bile salts like taurocholate and cholate, on *C. difficile* spore germination, was documented over 4 decades ago, and taurocholate has since been used as a standard method to enhance spore recovery and improve sensitivity for use in patient diagnostics [49,51]. Conversely, the inhibitory effect of another primary bile salt chenodeoxycholate (CDCA) and its analogues, as well as muricholic bile salts on *C. difficile*, has been shown in both humans and mice [52,53]. CDCA inhibits taurocholate-mediated spore germination in a competitive manner, with *C. difficile* spores demonstrating a higher affinity for CDCA than taurocholate [53]. Whilst *C. difficile* spores rely on relative concentrations of various bile salts as prompts for their germination, microbiota species responsible for producing secondary bile salts are also key players in determining CDI outcome as they are potent inhibitors of *C. difficile* vegetative growth *in vitro*. Bile salts like lithocholate and its epimers selectively prevent *C. difficile* growth, whilst others like taurine-conjugated ursodeoxycholate can block *C. difficile* toxin-mediated host cell apoptosis [54,55]. Interestingly, the secondary bile salt hyodeoxycholate can enhance spore germination at low concentrations in a dose-dependent manner [56]. Furthermore, distinct bile salt profiles have been associated with *C. difficile* first-time and recurrent infections. The former are characterized by significantly lower levels of the secondary bile salts, lithocholate and deoxycholate, whilst recurrent infections are associated with significantly higher levels of primary bile salts [57].

*Clostridium scindens* is one of the well-documented commensal species that employs a secondary bile salt-dependent mechanism of inhibition of *C. difficile*. This inhibition is attributed to its 7-alpha dehydroxylating activity which is encoded in a bile acid inducible (*bai*) operon [15,58]. The *bai* operon encodes an elaborate multistep pathway comprising a series of oxidation reactions followed by reductive steps to produce secondary bile salt end products. This pathway, beginning with the deconjugation of primary bile salts by bile salt hydrolases (BSHs), followed by their conversion into secondary bile salts through a -alpha dehydroxylation process, ensures that secondary bile salts that are inhibitory to spore germination remain in high concentration in the gut [59,60]. Other *Clostridium* species such as *Clostridium hiranonis* and *Clostridium leptum* that encode orthologues of the *bai* operon were also shown to inhibit *C. difficile* and protect against infection [61,62]. As such, BSHs encoded by microbiota species may also impact CDI outcomes. A recent study reported that a diverse range of BSHs encoded by gut commensals within *Lactobacillaceae* contribute to shaping the pool of conjugated bile salts and prevent *C. difficile* spore germination and virulence [63]. Interestingly, *C. difficile* was also shown to possess BSHs that deconjugate taurine-conjugated bile salts to generate cholate [64].

Although secondary bile acid salts produced by the gut microbiota strongly influence CDI, bile salt-independent mechanisms do exist. Members of the microbiota have been shown to suppress *C. difficile* virulence via other mechanisms. *Bacteroides thetaiotaomicron* cell wall glycans have been linked to the suppression of toxin release via the inhibition of autolysis, *in vitro* [65]. Additionally, the secretome of *C. scindens* has been shown to suppress *C. difficile* virulence genes *tcdA* and *tcdB* in a manner independent of bile salt metabolism [66], illustrating the multifactorial inhibitory nature of many of these species. It has also been noted that 7-alpha dehydroxylation by bacteria such as *C. scindens* is dispensable and not necessary for protection against *C. difficile in vivo*, *s*uggesting that high levels of secondary bile salts may act more as a biomarker for *C. difficile* invasion resistance and suggest that colonization resistance against CDI may occur largely independently of bile salt-associated mechanisms [62]. Furthermore*,* sublethal concentrations of bile acids produced by *C. scindens* were reported to enhance biofilm formation by *C. difficile*, indicating that microbiota-mediated bile acid levels may modulate *C. difficile* persistence in the gut [67].

### Amino acids and bacterial competition

Competition and availability of specific amino acids (aa) and nutrients are critical for *C. difficil*e growth. aa like proline and glycine are essential for *C. difficile* growth through Stickland metabolism, and *C. difficile* Stickland enzyme activity is also considered to be a biomarker for CDI [68,69]. This fermentation, primarily performed by *Clostridia* species, involves the reduction and oxidation of specific pairs of aa to produce ATP [70]. Glycine acts as a preferential co-germinant, promoting spore germination, and has been implicated in *C. difficile* virulence [71,73]. *In vivo* mouse models demonstrate how high levels of free proline increase during dysbiosis, creating a prime environment for *C. difficile* proliferation and infection [17]. In contrast, *C. leptum*-, *C. scindens*- or *C. hiranonis*-colonized mice were depleted of proline and were protected against CDI [62]. The importance of proline was further highlighted as a *C. difficile* mutant that is unable to utilize proline, lacking the d-proline reductase A (*pdrA*) gene, was unable to colonize and effectively establish infection, irrespective of whether mice had dysbiosis or a healthy microbiota present [17]. It was also shown *in vitro* that the ability of *C. difficile* to benefit from proline fermentation is contextual and largely dependent on microbiota composition, with some commensals driving its reliance on proline fermentation and others rendering it less beneficial [74]. Co-culturing *C. difficile* along with *Clostridium xylanolyticum* and *Paeniclostridium* spp. seemed to promote reliance on fermentation, whilst *C. scindens* did not [74]. This concurs with the finding that *C. scindens* inhibits *C. difficile* via its own proline-dependent Stickland metabolism, reducing the proline bioavailability and hence impeding its growth [62,68]. Thus, proline is clearly an important aa in *C. difficile* metabolism and growth.

The SCFAs propionate, butyrate and acetate play a crucial role in modulating colonization resistance in the gut [75]. The effect of butyrate on *C. difficile* has been well studied, although not entirely clear. Butyrate has been associated with reducing growth and enhancing CDI clearing in a murine model of CDI [76]. It also reduces *C. difficile* toxin-associated inflammation through changes in the production of pro- and anti-inflammatory cytokines and the intestinal permeability of the gut [77]. However, studies have also reported that butyrate induced a dose-dependent increase in sporulation and toxin production *in vitro* [78]. Interestingly, an *in vivo* bi-association study with *B. thetaiotaomicron* showed that *C. difficile* shifts its metabolism, converting commensal-derived succinate to butyrate to promote its expansion and infection in a perturbed gut [13]. It is important to note that butyrate is significantly reduced in symptomatic CDI patients, suggesting a protective role for this metabolite [76]. As the *in vitro* data shows butyrate inhibition is dose-dependent, a possibility is that the relatively small increase generated through the succinate-butyrate pathway may be sufficient for *C. difficile* survival. Moreover, *C. difficile* has also been shown to persist in the gut through the production of *para*-cresol, a bacteriostatic compound which provides a competitive advantage over other resident commensals; *para*-cresol can inhibit members of the *Bacteroidaceae* and *Enterobacteriaceae* families, promoting dysbiosis [79]. In addition to competing for nutrients, direct killing and toxin-degrading capabilities were reported for a newly characterized strain of *Bacillus velezensis* which specifically inhibits *C. difficile* colonization, with negligible impact on the local commensal microbiota community [80]. Thus, inter-species interactions could be useful tools for novel therapeutics against CDI [80,81].

Whilst inter-species competition plays a large role in perturbing CDI, competition between *C. difficile* strains has been reported. Interestingly, pre-colonization of one *C. difficile* strain provides protection from more virulent strains in both hamster and murine models [82,84]. This observation mirrored speculations surrounding the mystery of asymptomatic carriage commonly observed in infants, a phenomenon thought to arise from the higher proportion of non-toxigenic *C. difficile strains* compared to toxigenic ones in this population [85,86]. This protection by pre-colonization was shown to be due to intra-species competition for specific aa, resulting in depletion of the co-germinant glycine and inhibition of germination of a second more virulent *C. difficile* strain [87]. To this end, using non-toxigenic *C. difficile* strains as treatments for relapsed CDI as well as a preventative for primary and recurrent CDI has been documented and reviewed previously [88,90]. However, it is worth noting that supplementing media with aa including glycine and proline has actually been shown to decrease toxin yield in *C. difficile* [91]. Whilst *in vitro* models may not accurately mimic physiological conditions in the gut, it nevertheless highlights a complex relationship between metabolism, growth and virulence.

### Secreted antimicrobial factors

Secreted factors can be a crucial determinant in shaping the ecological architecture of the gut. The bacteriocin thuricin CD is produced by *Bacillus thuringiensis* and can inhibit a range of *C. difficile* clinical isolates [92]. More recently, specific isolates of *Lactobacillus agilis* and *Clostridium butyricum* were shown to produce extracellular antimicrobials effective against *C. difficile* [93]. From a recent screen of the probiotic potential of various *Lactobacillus* spp., *Lactobacillus reuteri* was shown to metabolize glycerol to reuterin, an antimicrobial compound with broad activity against commensals, with *C. difficile* showing high sensitivity *in vitro* [94]. *C. difficile* killing was demonstrated to be reuterin-dependent *in vitro* and subsequently shown to protect against toxin-mediated epithelial damage *in vivo* and induce reactive oxygen species production in *C. difficile* [95]. Nisin, a bacteriocin produced by *Lactobacillus lactis,* is known to have activity against a range of Gram-positive pathogens, including *C. difficile* [96,97]. Nisin also significantly reduces *C. difficile* spore germination *in vitro* [98]. A recent *ex vivo* colon model study showed nisin effectively killed *C. difficile*, but that it also induced compositional changes to the faecal microbiota, decreasing alpha diversity in a dose-dependent manner [99]. The bias towards Gram-positive activity was also shown by Le Lay *et al.* in a human gut model, showing effective inhibition of *C. difficile* at sufficiently high concentrations with minimal perturbations to the microbiota [100].

### Beneficial interactions

Interactions between commensal species and *C. difficile* can also be beneficial to the pathogen. *Enterococcus faecalis* was shown to promote both *C. difficile* fitness and pathogenesis in dual-species biofilms, showing increased toxin production and vancomycin resistance and aiding pathogenesis through metabolic remodelling of the gut milieu by cross-feeding specific fermentable aa [101]. Enterococci and *C. difficile* were also shown to co-localize in the lumen and form biofilm-like aggregates, suggesting that the biofilm architecture may help enhance the antibiotic protection seen and promote infection persistence [101]. Metal sequestering, mediated by the gut species *Desulfovibrio piger*, was shown to enhance tolerance of *C. difficile* to metronidazole [102]. Interestingly, *C. difficile* was also reported to aggregate with the mucus-associated microbiota member *Fusobacterium nucleatum* through the *F. nucleatum* adhesin RadD, which resulted in enhanced *C. difficile* biofilm formation [103]. Moreover, *C. difficile* can manipulate the resident gut microbiota to produce indole, an antimicrobial molecule, which helps to prevent the restoration of the protective microbiota during infection, prolonging persistence [104].

## *C. difficile* interactions with the host

*C. difficile*–host studies have largely focused on interactions between *C. difficile* toxins or other virulence factors and the host, and such interactions have been extensively reviewed elsewhere [105,106].

### Toxin interactions with host receptors

The primary glucosyltransferase toxins A (TcdA) and B (TcdB) bind to their respective receptors on human colonocytes, initiating a chain reaction of events which results in the inactivation of Rho-family GTPases and disruption of the host cytoskeleton and leading to a loss of epithelium integrity. A third *C. difficile* binary toxin (CDT) catalyses the depolymerization of actin and leads to microtubule protrusions that enhance its adhesion [107]. CRISPR-Cas screens have recently shown that sulphated glycosaminoglycans and low-density lipoprotein receptors contribute to the binding and entry of TcdA [108]. Wnt receptor Frizzled protein and chondroitin sulphate proteoglycan 4 have been shown to be receptors of TcdB and are exploited by *C. difficile* to promote epithelial barrier disruption [109,110]. More recently, tissue pathway inhibitory protein (TFPI) has been identified to be a colonic crypt receptor for toxin B variants from clade 2 strains which cause more severe infections [111]. The binary toxin, CDT, produced by select epidemic strains such as ribotypes 027 and 078, was shown to bind to the lipolysis-stimulated lipoprotein receptor, with truncation experiments showing it is highly effective, requiring only 109 bp of CdtB to bind sufficiently [112]. Whilst the toxins are important virulence determinants, there are several other host factors that determine the success of this pathogen.

### Host metabolites and metal ions

Host- or diet-derived metabolites and metal ions play a key role in aiding the survival of *C. difficile*. Ornithine, a host-derived aa, which is either converted to proline via a reductive pathway or which undergoes an oxidative Stickland reaction to yield acetate, ammonia and alanine, has been shown to maintain *C. difficile* during asymptomatic infection [113]. *C. difficile* is also adept at manipulating host factors to its benefit: whilst it utilizes the host-derived metabolite sorbitol during CDI *in vivo* to aid its persistence, it also induces host aldose-reductase activity responsible for converting glucose to sorbitol through its toxins [114].

Metal ion limitations can also impede pathogen fitness [115]. Calprotectin, a zinc (Zn)-binding protein, impacts *C. difficile* growth by limiting Zn availability and is considered an important determinant in protecting against CDI and limiting its severity and recurrence [16]. ZupT, a Zn transporter used by *C. difficile* to survive calprotectin-mediated metal limitation, was employed by *C. difficile* to survive and protect against such growth inhibition [116]. Calprotectin-mediated Zn limitation is also thought to limit *C. difficile* metabolic activity, shifting its metabolism from proline Stickland fermentation to mannitol, to sustain its own colonization, highlighting how *C. difficile* can adapt its metabolism to the dynamic host environment [117]. *C. difficile* also employs several mechanisms to obtain iron, another essential element, including utilizing iron from siderophores like ferrichrome produced by other microbes [118] and formation of ferrosomes – bacterial membrane-bound organelles that store iron by a biomineralization process [119]. Heme is the other host component that *C. difficile* sequesters through the hsmRA system, which enables its effective use in protection from oxidative stress in the gut [120].

### Host antimicrobial peptides

Antimicrobial peptides (AMPs) are a diverse group of peptides produced by both host and microbiota that are vital for the microbiome homeostasis and facilitate cross-communication [121]. They exhibit broad-spectrum antimicrobial activity, restricting the microbiota population and preventing pathogen colonization. AMPs are produced by different epithelial lineages, largely by enterocytes and Paneth cells, and can be controlled by bacterial signals in the gut [122]. AMPs or host-defence peptides are actively being considered as a desirable treatment approach for CDI [123,124], and various AMPs such as LL-37 [125], NVB302 [126], NCK-10 [127], surotomycin [128], and rampoplanin [129] have demonstrated robust activity against *C. difficile*.

The host cathelicidin LL-37 and the human defensin HBD3 result in depolarization of vegetative *C. difficile* cells and significantly lowered *C. difficile* numbers *in vitro*. HBD3 was also shown to disrupt the cell wall of *C. difficile* [125]. However, sensitivity to LL-37 was shown to be *C. difficile* strain-specific, with the hypervirulent endemic 027 ribotype demonstrating the greatest resistance [130]. Similar phenotypes have been demonstrated *in vivo*; exogenous addition of LL-37/CRAMP was shown to attenuate *C. difficile*-associated colitis in a murine model [131]. The effect of host AMPs on *C. difficile* toxins was shown by Giesermann *et al*. who demonstrated that treatment with alpha-defensins but not LL-37 or beta-defensin inhibited toxin B [132]. Indeed, *C. difficile* can adapt to AMPs by tuning its transcription through regulators like ClnrAB [133] and/or by encoding cationic AMP (CAMP) resistance genes [134]. However, synergism of AMPs with antibiotics has been demonstrated, whereby combinations of the AMPs HBD3 or LL-37 with antibiotics resulted in more potent *C. difficile* killing [125], suggesting future potential for novel therapeutic cocktails.

## Three-way crosstalk

**Fig. 2.**
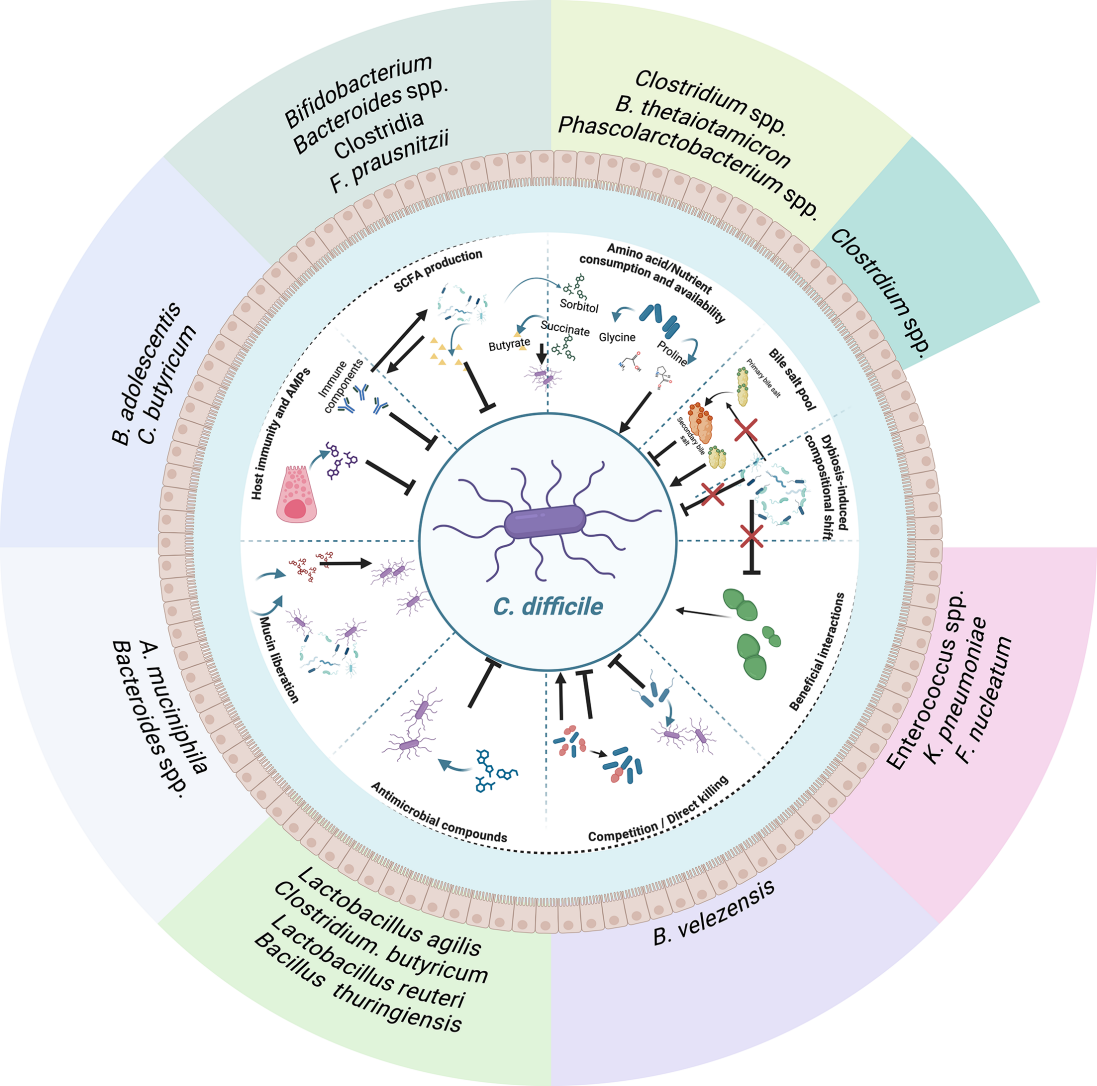
Three-way interactions between the gut microbiota, *C. difficile* and the host. The microbiota can mediate interactions between both host and *C. difficile*. Known interactions between *C. difficile* and commensals can be both inhibitory and beneficial to *C. difficile*. Many commensal species produce SCFA, which can either be inhibitory to *C. difficile,* commonly with high levels of butyrate. Such fatty acids can also modulate the host immune system and can induce T-cell differentiation and induction of IL-17 and IL-22, which in turn can impact *C. difficile* proliferation and growth. Commensals also play an important role in shaping the bile salt pool, which can inhibit or stimulate *C. difficile* spore germination. Loss of the normal microbiota during dysbiosis leads to enterococcal enrichment which has been associated with enhanced *C. difficile* pathogenesis. Liberation of Stickland substrates by various host mucin degraders provides substrates that *C. difficile* competes for, and it often switches its metabolism away from fermentation depending on the gut composition. Loss of commensals and associated competition allows *C. difficile* to thrive on the Stickland substrate-rich environment. The link between the immune system, metabolites and *C. difficile* is also apparent; IL-22 signalling can promote the growth of *Phascolarctobacterium* spp. which can consume sorbitol, which is a necessary metabolite for *C. difficile,* therefore inhibiting it. Both the host and microbiota members can produce inhibitory antimicrobial compounds which not only can inhibit *C. difficile* but also various other pathogens. Figure was generated using BioRender.com.

It is evident that many aspects of host*–C. difficile* interactions in the gut are likely to be influenced, driven or modulated by both the microbiota and host factors. It is therefore pertinent to attempt to bring the three players together: host, pathogen and microbiota (Fig. 2). A few examples of such three-way interactions are presented below.

### Mucins

A major contributor in ensuring homeostasis between microbiota and host is the mucus which lines the gut. The intestinal mucus is comprised predominately of mucins, mainly MUC2, and provides both an interface and a barrier between luminal contents and host [135,136]. Mucins, which are a rich source of glycans, as well as many Stickland substrates such as proline, threonine and serine, provide an ample resource for *C. difficile* fermentation, aiding its proliferation [137]. Although *C. difficile* lacks glycosyl hydrolases necessary to degrade mucin glycans, it can move towards them via chemotaxis and adhere to MUC2 glycans and their oligosaccharide derivatives [138]. To overcome this enzymatic shortcoming, *C. difficile* relies on the array of mucin-degrading bacteria found within the microbiota, which harbour the glycosyl hydrolases necessary to cleave glycan linkages [139]. *Akkermansia muciniphila* is the best-known ‘mucin specialist’ capable of hydrolysing 85% of mucin structures, and *Bacteroides* spp. are known as generalist degraders capable of switching readily from degrading dietary glycans to mucin glycans due to their vast arsenal of glycosyl hydrolase enzymes [139]. In CDI patients, the mucus itself undergoes a compositional shift, characterized by decreased levels of MUC2 and a shift towards MUC1 as the primary mucin. This is accompanied by acidification of the mucus and alterations in specific oligosaccharide residues such as galactose [140]. Engevik *et al*. also demonstrated reduced MUC2 expression in both a human intestinal organoid model injected with *C. difficile* and stool samples from CDI patients [140]. TcdA is known to bind to certain galactose residues which can be found on many proteins on erythrocytes, as well as human IgG [141,142]. It is possible that this CDI-induced shift may make the environment more favourable for toxin binding. This network between host-derived mucins, their degradation by microbiota or toxin-mediated disassembly and their subsequent acquisition by *C. difficile* highlights a nuanced three-way link between microbiota, pathogen and host and showcases how *C. difficile* can exploit the activity of commensals to scavenge nutrients and remodel the mucin architecture for its own benefit.

### Host immune cells and factors

Immune cell interactions of *C. difficile* are an important element of CDI, and there is growing evidence that the immune system can play a multifaceted role during infection, promoting or protecting against disease progression [143]. The host immune system relies upon the microbiota for protection [144], and the crosstalk between host and microbiota is key to influencing the immune response. Interactions between host and microbiota have been associated with various aspects of immune modulation and T-cell maturation and differentiation, intestinal barrier maintenance and host colonocyte metabolism [145].

IL-17 and IL-22, cytokines produced by T helper 17 cells that are important for maintaining intestinal barrier integrity, are induced by commensals like *Bifidobacterium adolescentis* [146]. IL-17 and IFN-γ and neutrophils were all required for the protective effect against CDI induced by the butyrate-producing commensal, *C. butyricum* [147]. IL-22 signalling was shown to regulate host glycosylation, permitting the growth of *Phascolarctobacterium* spp. which consumes succinate, a necessary metabolite for *C. difficile* [148]. Moreover, IL-22 has been associated with limiting translocation of commensals that can act as pathobionts into circulation during CDI through regulating the complement-system regulation and thus reducing mortality during CDI in mice [149]. Antibiotic treatment and high-fat diets are known to be associated with a similar increased commensal translocation and dysregulation of immune cells [150,151]. Thus, antibiotic-induced shifts in the microbiota can provide a suitable niche for *C. difficile* to proliferate and, along with dietary effects, could also contribute to increased inflammation and enhanced pathobiont effects during CDI.

The metabolic environment of the gut is key in defining the microbiota composition as well as the local immune responses. Recently, a eukaryotic commensal, *Tritrichomonas musculis*, was shown to control CDI pathogenesis through actively metabolizing arginine, reshaping the microbiota and creating a low ornithine environment that alleviates CDI [152]. *T. musculis* also reduces tissue damage caused by neutrophils by inhibiting their recruitment which reshapes the local immune response, ultimately resulting in an increase in production of mucus [152]. Other commensal gut bacteria like *Faecalibacterium prausnitzii* produce SCFAs like propionate, butyrate and acetate from the breakdown of complex carbon sources, which are immunomodulatory, playing crucial roles in the regulation, recruitment and differentiation of immune cells, as well as the regulation of proinflammatory cytokines [153].

The link between host, microbiota and pathogen is further evident with the use of FMTs. FMT-mediated resolution of CDI was shown to depend on specific adaptive cell subsets; CD4+ regulatory T-cells but not CD8+ T cell or B cells were demonstrated to be important for the efficacy of FMTs [154]. Furthermore, patients with recurrent infections present with lower frequencies of CD4+ Th17 cells, and FMT treatment was shown to enhance TcdB-specific Th17 cells [155]. FMTs have also been shown to increase colonic IL-25, a microbiota-linked cytokine which protects from CDI through controlling numbers of eosinophils [18]. Additionally, FMTs are known to restore systemic immunity and rescue pathogen-mediated sepsis in mice via an IFN regulatory factor-3-dependent mechanism of pathogen clearing, characterized by expansion of butyrate-producing Bacteroidetes [156]. Thus, the immune status of the host is clearly important for the success of microbiota-based treatments for CDI.

A significant limitation in our current understanding of *C. difficile*–host–microbiota interactions is the heavy reliance on simplified experimental systems. Many studies examining the ability of *C. difficile* to adhere to MUC2, co-localize with the mucus layer or undergo transcriptomic shifts in response to mucus [103,157], have used *in vitro* models, using simpler cell lines or organoids. Whilst these systems offer valuable insights and circumvent the invasiveness and ethical concerns of *in vivo* methods, they lack the complexity of the native gut environment. Moreover, many commensals possess an extensive repertoire of glycosyl hydrolases capable of degrading host mucins and glycans for energy [139]. However, although the potential for microbiota-mediated mucin catabolism is clear, the specific activity and dynamics of these enzymes, particularly during active CDI, remain poorly characterized. Indeed, there is also a widespread reliance on murine models for *in vivo* studies. Whilst such models allow interrogation of host–immune interactions, it is important to acknowledge differences between different whole animal models. For instance, discrepancies in the roles and essentiality of *C. difficile* toxins A and B have been reported between hamster and mouse models [158,160]. Even in humanized gnotobiotic mice, only a subset successfully replicates the donor’s microbial phenotype [161], highlighting inter-individual variability and the limitations of extrapolating findings to human disease. Taken together, these issues introduce a degree of uncertainty that complicates efforts to disentangle the specific contributions of host, microbe and pathogen within this intricate tripartite relationship. As such, whilst current models continue to yield valuable insights, generalizations should be made with caution and validated across multiple model backgrounds.

## Studying three-way interactions

Many of the studies into microbiota–pathogen–immune cell interactions have been performed using murine models of CDI and have provided great insight into complex interactions. Recent work has also employed gnotobiotic mice with human-derived microbiota to study recurrent CDI [162]. However, in addition to having the limitations of murine microbiota and immune system, and the ethical issues associated with animal models, such models do not enable studies at a cellular or single-cell level. Additionally, studying dynamics or visualizing the sequence of events and interactions is challenging.

Several in *vitro* gut systems have been described including batch fermenters, compartmentalized systems or continuous culture fermentation models used to simulate different environments within the gut [163,165]. A Simulation of the Human Intestinal Microbial Ecosystem (SHIME) model, an example of a multi-compartmental system, was developed further to include a mucosal environment using mucin-covered microcosms (M-SHIME), facilitating a more *in vivo-like* simulation [163,164]. A benefit of the continuous models is that longitudinal studies can be performed with relative ease, allowing investigations into dynamic microbiota interactions [163,164]. A model that has been widely used for *C. difficile* and *C. difficile*–microbiota interactions studies is the triple-stage chemostat model which mimics the proximal, mid and distal human colon environments [166]. This model uses pooled faecal slurries and has been used to study the impact of various antibiotics on *C. difficile*, as well as to study the role of biofilms in recurrent infection [167,170]. To address the challenges associated with multi-stage fermenters, which are often labour-intensive, technically demanding and lack scalability, the miniaturized multi-stage *in vitro* model, MiGut, has shown significant promise. This model offers a scalable alternative whilst maintaining a high correlation with previously established and validated *in vitro* gut models [171].

Other *in vitro* biofilm models have been developed where gut commensal species can be tracked within a multi-species microbiota community over time [172]. However, a major drawback in these models is the lack of inclusion of mammalian cells, which is necessary to study the complex gut environment, given the important role of the host responses during CDI.

### Two-dimensional and three-dimensional models

Monolayers of various cancer-derived cell lines have been widely used to study host–bacterial interactions. Typically, polarized colonic epithelial cell lines like Caco-2, HT-29 or T84 have been used to mimic the tight epithelial barrier, and the interactions between host cells and *C. difficile* toxins have been studied extensively using these cell lines [106]. *C. difficile* vegetative cell adhesion mediated by adhesins like Cwp2 [173] and adhesion of spores [174] has been demonstrated using such co-cultures of whole bacteria with epithelial cells. Whilst cell lines offer a simple, economical and readily reproducible approach, they do not represent the complex physiology of the gut epithelium. Three-dimensional models offer a model more reminiscent to the native gut, and gut organoid models developed in recent years have been employed for studying enteric infections [175]. Vegetative *C. difficile* microinjected into a human intestinal organoid model was shown to survive for 12 h, with toxins produced disrupting the epithelial barrier [176]. Spore germination and toxin damage by *C. difficile* were also demonstrated in a human three-dimensional bioengineered intestinal tissue model [177]. Other two-dimensional human enteroid models have also been developed which enable imaging of the changes induced by CDI up to 14 h [178]. A major drawback of these models is that as there are no environmental controls, infection time frames are limited to 12–14 h.

Vertical diffusion chamber (VDC)-based CDI models, which offer better environmental control with a dual aerobic-anaerobic environment, have been recently reported and enable longer co-cultures (up to 48 h) [179,180]. The VDC systems typically have aerobic and anaerobic chambers which enable the co-culturing of both intestinal cells and fastidious anaerobes in tandem [179]. Anonye *et al*. reported adhesion of *C. difficile* strains to two-dimensional and three-dimensional bioengineered epithelial layers containing Caco-2 and HT29-MTX mucus-producing cells and CCD-18Co myofibroblasts and formation of microcolonies over 48 h. Production of cytokines such as IL-8 has also been shown. A dual RNA-seq analysis approach utilized this infection model to identify temporal transcriptional changes occurring in both the gut cells and *C. difficile* during early infection [181]. This model has also been used to demonstrate the inhibitory effects of commensals like *Bacteroides dorei* on CDI. However, being a static system with no replenishment of nutrients, longer time frames of infection, for example, to model bacterial persistence, are not possible.

Mozarati *et al*. developed a two-compartment Host-Microbiota Interaction (HMI) module, integrating SHIME with an enterocyte layer and a mucosal area for bacterial adherence, separated by a semipermeable membrane. This model can have either semi-continuous or continuous flow [182]. An oxygen gradient across the epithelium similar to *in vivo* conditions was demonstrated with cell viability exceeding 48 h following microbial community exposure [182]. The HuMix (human microbial crosstalk) model comprising co-laminar microchannels with oxygen sensors was then developed which allows for cocultures of anaerobic commensals with multiple cell lines such as Caco-2, myofibroblast CCD-18Co and primary CD4+ T cell lines [183]. Gut-on-a-chip models, which utilize cell lines of organoid-derived cells, employ microfluidic chips and offer further refinement of physiological conditions and the gut epithelial architecture by replicating environmental as well as mechanical cues in the intestinal environment such as mechanical stretching and shear stress [184]. Several such intestine-on-a-chip devices have successfully been used to culture obligate anaerobes like *Bacteroides fragilis*, as well as microbiota communities [185,187]. Furthermore, the immune cells, critical players at the gut-microbial interface, were included in a recent gut epithelium-microbe-immune micro-physiological system which enabled the continuous co-culture of *Faecalibacterium* spp. alongside a colonic epithelium, antigen-presenting cells and CD4+ naïve T cells [188].

Although there is a fast pace of development of new models, their application to targeted commensal–host or host–commensal–pathogen interaction studies has been limited. Accessibility and availability of expertise to run some of the sophisticated microfluidic systems, along with high associated costs, have likely limited their wider use in gut microbiology.

### *In silico* modelling

Over the last few decades, *in silico* approaches are becoming increasingly attractive for examining complex microbial interactions as either standalone approaches or in tandem with experimental data that is often ‘multi-omic’.

The widely used generalized Lotka–Volterra (gLV) model has been employed to predict community dynamics as a function of growth. Such models have been beneficial in predicting both bacterial co-existence and community structure [189] and can predict interactions well even in complex environments [190]. Alterations to the conventional gLV model have generated the ‘compositional’ Lotka–Volterra (cLV) model, an alternative linear model that can accurately predict temporal community dynamics [191], with high correlation between observed microbiome dynamics [192]. Moreover, cLV has been employed to predict *C. difficile* concentrations based on murine infection data [191]. cLV models have also been used to probe community-level functional activity, allowing the prediction of metabolite concentrations based on species abundance [193]. However, as simpler models may not adequately capture certain types of interactions [194,195] and are often limited to single time-point predictions, the overall use of an LV-based model for community approximations remains somewhat disputed [190,196]. However, in the context of modelling active CDI, where multiple significant environmental changes occur, employing gLV alone is unlikely to capture community-level behaviours due to the mathematical parameters and constraints of this model [197].

Neural networks, such as recurrent neural networks (RNNs), are universal function approximators, used to model dynamic systems [198]. Although such networks provide greater flexibility than gLVs, the parameter requirements are often higher, which can often make model fitting and generalizability challenging [196]. Nevertheless, RNNs have been shown to not only outperform gLVs but have allowed the construction of synthetic communities and have been used to model dynamic time-dependent changes in species abundance and metabolite production in a diverse 25-member synthetic community [196]. This study demonstrated successful prediction of species abundance at five discrete time points over a 60-h period, based only on initial species abundance. It also identified differing roles in some of the key microbial community players, highlighting that metabolite production is significantly driven by *Actinobacteria*, *Firmicutes* and *Proteobacteria*, whilst *Bacteroides* shape the community dynamics by modulating species growth and overall community architecture [196]. Another neural network fragment, MiMeNet, was not only able to make better metabolite predictions compared to linear models but was also able to identify underlying interaction patterns to delineate microbe–metabolite interaction networks [199].

### Genome-scale metabolic models

Given the strong influence metabolism has on inter-microbial and host-microbial interactions, modelling of microbial communities using metabolic genome-scale metabolic models (GEMs) is rapidly becoming a popular approach to probe host–microbe, microbe–microbe and diet–microbiome interactions under specific physiological conditions [200]. These are computational constructions of the metabolic capacity encoded by the genome of any given organism and can be used to predict metabolic capacities of specific gut bacteria and rates of metabolite production and consumption. The growth rate of the specific organism can then be predicted by simulating the metabolic fluxes within the model system, using methods such as the classical flux balance analysis (FBA), or its derivatives 13C-metabolic flux analysis and dynamic FBA [201]. These models can be further contextualized through the integration of multi-omics data, and such conjunctions have been used to study dynamics in both non-disease and disease states in various microbiome backgrounds [202]. This allows the resulting FBA, for example, to better reflect the experimental conditions of interest. Many GEMs are available on various databases such as the virtual metabolic human (VMH) database [203]. Originally comprising over 800 different microbe GEMs [203], following the recent implementation of an updated version of the Assembly of Gut Organisms through Reconstruction and Analysis (AGORA) reconstruction resource (AGORAV2), the VMH has been updated to contain 7,302 human micro-organism strains [204], providing a wealth of resource for researchers.

Most approaches to generate context-specific GEMs rely on the integration of transcriptomic data, mostly through constraining fluxes of reactions associated with genes with low/high transcript abundances based on user-defined cutoffs with the aid of different algorithms [205,210]. An alternative approach combines transcriptomic abundances and flux parsimony and reaction pruning to identify the most cost-effective metabolic usage that reflects the cell’s transcriptional investments [211]. Taking 16S rRNA data and investigating community metabolism from patients with recurrent *C. difficile* and employing *in silico* pipelines and GEMs revealed higher *Enterobacteriaceae* abundance, with a predicted reduction in secondary bile salt synthesis and enhanced aromatic aa catabolism, predicting *in silico* that it creates a favourable milieu for spore germination [212]. Combining the integrations of transcriptomic data with genome-scale models has been used to show mucin-driven shifts in metabolism of *Pseudomonas aeruginosa* [213]. With *C. difficile*, such tools have revealed its reliance on specific pathways like the pentose phosphate pathway, as well as increased metabolite usage like cytidine and *N*-acetylneuraminate when expression of virulence factors is reduced [214]. Moreover, transcriptome-integrated *C. difficile* GEMs have identified strain-specific differences in metabolism that differ between high and low toxin expression, giving further insight into *C. difficile* pathogenesis [215]. Whole-community metabolic modelling demonstrated how *Enterobacteriaceae* may create a favourable milieu for *C. difficile* colonization [216]. Simulating a gut community has also been done to elucidate metabolic interactions of the gut microbiota in different disease states such as type 2 diabetes [217]. Here, GEMs of select gut microbes associated with type 2 diabetes were integrated to create a community metabolic model highlighting how researchers can start to probe more disease-related questions in a tailored *in silico* gut community.

## Conclusions

It is evident that the microbiota plays a pivotal role in protecting against CDI through different strategies, including controlling metabolites essential for its growth, producing antimicrobials, stimulating the host immune system and ensuring the gut environment is inhospitable to spore germination. *C. difficile* employs many counter tactics to the assault from the host and microbiota; it can manipulate its metabolic profile as well as modulate its growth stage, shifting towards sporulation to enable its survival. Protection against this pathogen ultimately appears to be a cumulative effect of several individual host and commensal-mediated mechanisms which depend on the composition of the microbiota and the host immune status. Clearly, there is a need to understand the inter-bacterial interactions within the microbiota and the specific host proteins that influence them, so that effective microbiome-based therapeutics can be designed. There has been development of a range of innovative *in vitro* laboratory models which have excellent potential to tease apart the intricate interactions between *C. difficile*, microbiota and host. Additionally, with the complexity of factors that can affect outcomes of these interchanges, rapidly developing enhanced *in silico* modelling tools will pave the way for precise predictions of multi-faceted interactions. Whilst important challenges remain such as strain-specific variability, inconsistencies between model systems and issues in extrapolating findings to humans, rapidly evolving technological and methodological advances offer promising avenues to bridge the current gaps in our knowledge.

## Abbreviations

AA: Amino acid
AGORA: Assembly of Gut Organisms through Reconstruction and Analysis
AMPs: antimicrobial peptides
bai: bile acid inducible
BSHs: bile salt hydrolases
CAMP: cationic antimicrobial peptide
CDCA: chenodeoxycholate
CDI: *Clostridioides difficile* infection
CDT: *Clostridioides difficile* binary toxin
cLV: compositional Lotka–Volterra
FBA: flux balance analysis
FMT: faecal microbiota transplant
GEM: genome-scale metabolic model
gLV: generalized Lotka–Volterra
HMI: Host-Microbiota Interaction
HUMIX: human microbial crosstalk
PMC: pseudomembranous colitis
RNNs: recurrent neural networks
SCFA: short-chain fatty acid
SHIME: Simulation of the Human Intestinal Microbial Ecosystem
VDC: vertical diffusion chamber
VMH: virtual metabolic human
